# Reports on One Thousand Soldiers Convalescent from Diseases of the Dysentery and Enteric Groups

**Published:** 1918-05

**Authors:** 


**Reports on One Thousand Soldiers Convalescent from Diseases of the Dysentery and Enteric Groups.** By WILLIAM FLETCHER, Captain, R. A. M. C. Abs. from the *Journ. R. A. M. C.*, Vol. XXX, N° 1, p. 51, Jan. 1918.

Eighteen carriers of the mannite-fermenting dysentery bacillus were found by direct plating in Endo's medium out of a total of 800 convalescents from the western front. In the first examination the bacillus was isolated in 12 cases; in the second examination it was found in 4 cases; and in the third, in 2 cases. On an average, not more that 4 1/2 weeks had elapsed from the date of onset to the time when the bacillus was isolated. In only three cases were the bacilli found more than once. In only one case were the organisms according to type. None of them was agglutinated to the end titer of the specific sera of the Flexner and Y types. All strains were agglutinated by low dilutions of sera specific for type strains of mannite-fermenting organisms and also, in most instances, by the patient's own blood serum in dilutions of 1/20 to 1/80. Most of the organisms were more like the Y type than the Flexner type.

One strain (1033) fermented saccharose, maltose, and dextrin in the first subcultures. This was the only one fermenting maltose when first isolated. Two others (206 and 479) acquired the power three months later. Six other strains (206, 479, 697, 794 and 1057) were able to ferment dextrin after frequent subcultivation. The power of producing indol varied. Five strains did not produce it. In others it was present at first but was lost later. In some instances the reverse was the case.

Two specific sera were used in investigating the reactions : the Flexner type (D. F. ) with a titer of 1/4,000 and a serum of the Y type (Y 2) with a titer of 1/500.

Two strains only were agglutinated by the Flexner serum in dilutions as high as 1/1,200. None was agglutinated by the Y serum diluted above 1/400.

When sera of the Flexner and Y type swere saturated with emulsions of the mannite-fermenting organisms which had been isolated, the specific agglutinins were removed by one strain only (794) from the Y serum. None of the cultures absorbed the specific agglutinins from the Flexner type serum.

Only one carrier of the Shiga type was detected. Five weeks after the beginning of the illness, Shiga colonies formed 2 o/o of all those on the plate. Eleven days later 40 o/o were Shiga colonies. Since that time it has been found in 35 out of 61 examinations.

